# Supplementary material for: Research effort devoted to regulating and supporting ecosystem services by environmental scientists and economists
Source: PLoS One. 2021 May 28;16(5):e0252463. doi: 10.1371/journal.pone.0252463 (PMC8162671; doi:10.1371/journal.pone.0252463)
Supplement: S1 Table — Biophysical ecosystem processes and functions described as regulating and supporting ecosystem services a(Value of Nature to Canadians Study Taskforce 2017), regulating NCP categories b(Díaz et al. 2018), and their corresponding synonyms (processes and functions) used as keywords in a literature search strings in bibliographic searches. (PDF) [file pone.0252463.s001.pdf]

PLoS ONE

Electronic Supporting Information: S1 Table

Title: **Research effort devoted to regulating and supporting ecosystem services by environmental scientists and economists**

Authors: Andrew N. Kadykalo, Lisa A. Kelly, Albana Berberi, Jessica L. Reid, C. Scott Findlay

S1 Table. List of 15 regulating and supporting ecosystem services. Biophysical ecosystem processes and functions described as regulating and supporting ecosystem services [a](Value of Nature to Canadians Study Taskforce 2017), regulating NCP categories [b](Díaz et al. 2018), and their corresponding synonyms (processes and functions) used as keywords in a literature search strings in bibliographic searches.

**Ecosystem Service**	**Ecosystem Service Category[a]**	**Regulating NCP Category[b]**	**Literature Search String for Ecosystem Service (Processes and Functions)**
Air Quality Regulation	Regulating	Regulation of air quality	"air pollution removal" OR "air purification" OR "air quality improvement" OR "air quality regulation" OR "gas regulation"
Biological Control	Regulating	Regulation of detrimental organisms and biological processes	"biological control" OR "biocontrol" OR "biological regulation" OR "herbivore control" OR "herbivore regulation" OR "herbivore suppression" OR "insect control" OR "pest reduction" OR "pest suppression" OR "pest regulation" OR "predation service*" OR "prey suppression" OR "suppression of pest" OR "suppression of pests" OR "suppress insects*" OR "suppress pest*" OR "suppress weed*" OR "trophic cascade effect*" OR "weed control" OR "weed regulation" OR "weed seed predation" OR "weed suppression"
Carbon Sequestration	Regulating	Regulation of climate	"carbon accumulation" OR "carbon capture" OR "capture carbon" OR "carbon fixation" OR "fix carbon" OR "carbon mitigation" OR "carbon retention" OR "carbon sequestration" OR "sequest* carbon" OR "carbon sink" OR "carbon storage" OR "store* carbon" OR "carbon uptake"
Coastal & Storm Protection	Regulating	Regulation of hazards and extreme events	"attenuate storm*" OR "attenuate wave*" OR "coastal protection" OR "dissipate wave*" OR "reduce wave*" OR "reducing wave*" OR "shore protection" OR "shoreline protection" OR "storm protection" OR "storm surge protection" OR "wave attenuation" OR "wave dissipation" OR "wave reduction"
Disease Regulation	Regulating	Regulation of detrimental organisms and biological processes	"disease control" OR "disease regulation" OR "disease suppression" OR "pathogen control" OR "pathogen regulation" OR "pathogen suppression"
Drought Mitigation	Regulating	Regulation of hazards and extreme events	"drought control" OR "drought mitigation" OR "mitigate drought*" OR "drought recovery" OR "drought resilience" OR "drought risk reduction"
Erosion Regulation	Regulating	Formation, protection and decontamination of soils and sediments	"control of soil erosion" OR "erosion control" OR "erosion mitigation" OR "mitigate erosion" OR "erosion prevention" OR "prevent erosion" OR "erosion reduction" OR "erosion regulation" OR "regulate erosion" OR "reducing erosion" OR "reduced erosion" OR "sediment capture" OR "sediment control" OR "sediment retention" OR "retain sediment" OR "sediment stabilization" OR "sediment stabilisation" OR "stabilize sediment*" OR "stabilise sediment*" OR "shoreline stabilization" OR "shoreline stabilisation" OR "stabilize shoreline*" OR "soil conservation" OR "soil retention" OR "retain

			soil" OR "soil stability"
Flood Regulation	Regulating	Regulation of hazards and extreme events	"flood alleviation" OR "flood attenuation" OR "attenuate flood*" OR "flood control" OR "flood prevention" OR "flood mitigation" OR "mitigate flood*" OR "flood protection" OR "flood reduction" OR "flood regulation" OR "regulate flood*" OR "flood resilience" OR "flood risk reduction" OR "flood retention" OR "flood storage" OR "flood water retention" OR "floodwater retention"
Habitat Provision	Supporting	Habitat creation and maintenance	"habitat maintenance" OR "habitat provision" OR "habitat service*" OR "habitat ecosystem service*"
Nutrient Cycling	Supporting	Formation, protection and decontamination of soils and sediments	"nitrogen retention" OR "nutrient cycling" OR "nutrient filtration" OR "nutrient recycling" OR "nutrient retention" OR "phosphorous retention" OR "recycling of nutrient*"
Pollination	Regulating	Pollination and dispersal of seeds and other propagules	"berry weight" OR "fruit set" OR "fruit amount" OR "fruit mass" OR "fruit weight" OR "fruit retention" OR "nut set" OR "pollination" OR "pollinating" OR "pollinated" OR "pollen deposition" OR "pollen deposited" OR "pollen tubes" OR "seed set" OR "seed amount" OR "seed mass" OR "seed weight" OR "seed deficit"
Seed Dispersal	Supporting	Pollination and dispersal of seeds and other propagules	"diplochory" OR "dispers* seed*" OR "myrmecochory" OR "secondary dispersal" OR "seed dispersal" OR "seed* dispersed" OR "seed dispersion" OR "seed movement" OR "seed shadow*" OR "seed transport"
Soil Formation	Supporting	Formation, protection and decontamination of soils and sediments	"bioturbation" OR "soil development" OR "soil evolution" OR "soil fertility" OR "soil formation" OR "soil generation" OR "soil genesis" OR "pedogenesis" OR "peat formation"
Water Purification & Waste Treatment	Regulating	Regulation of freshwater and coastal water quality	"dung burial" OR "nitrogen removal" OR "purification of water" OR "waste treatment" OR "water filtration" OR "water purification"
Water Regulation	Regulating	Regulation of freshwater quantity, location and timing	"freshwater regulation" OR "hydrological regulation" OR "hydrological ecosystem services" OR "hydrological services" OR "soil moisture retention" OR "water cycling" OR "water flow regulation" OR "water quality maintenance" OR "water quality regulation" OR "water regulation"

References

Díaz S, Pascual U, Stenseke M, Martín-López B, Watson RT, Molnár Z, et al. Assessing nature's contributions to people. Science. 2018;359(6373): 270-272. doi: 10.1126/science.aap8826.

Value of Nature to Canadians Study Taskforce. Completing and Using Ecosystem Service Assessment for Decision-Making: An Interdisciplinary Toolkit for Managers and Analysts. Provincial, Federal, and Territorial Governments of Canada, Ottawa, ON; 2017.
Avialable from: http://biodivcanada.ca/default.asp?lang=En&n=B443A05E-1
