# Supplementary material for: Research effort devoted to regulating and supporting ecosystem services by environmental scientists and economists
Source: PLoS One. 2021 May 28;16(5):e0252463. doi: 10.1371/journal.pone.0252463 (PMC8162671; doi:10.1371/journal.pone.0252463)
Supplement: S2 Table — Ecosystems as broad ‘biomes’ or ‘ecoregions’ and ‘anthromes’ classes, which were further refined into finer-scale ‘subsystems’ and the corresponding keywords used in literature search strings in bibliographic searches for ecosystem type. Due to the diversity of inland wetland types, we employed both a general wetland search terms as well as terms based on higher resolution wetland classes. (PDF) [file pone.0252463.s002.pdf]

PLoS ONE

Electronic Supporting Information: S2 Table

Title: **Research effort devoted to regulating and supporting ecosystem services by environmental scientists and economists**

Authors: Andrew N. Kadykalo, Lisa A. Kelly, Albana Berberi, Jessica L. Reid, C. Scott Findlay

S2 Table. List of 32 ecosystem classes (whether subsystems, biome/ecoregion, or anthromes) here referred to as 'ecosystem types'. Ecosystems as broad 'biomes' or 'ecoregions' and 'anthromes' classes, which were further refined into finer-scale 'subsystems' and the corresponding keywords used in literature search strings in bibliographic searches for ecosystem type. Due to the diversity of inland wetland types, we employed both a general wetland search terms as well as terms based on higher resolution wetland classes.

Biomes/Ecoregions	Subsystem	Literature Search String for Ecosystem Type
Arctic and Mountain Tundra	Arctic Tundra	("arctic" OR "antarctic" OR "polar") AND (tundra)
	Mountain Tundra	("alpine zone") OR (("alpine" OR "mountain*") AND (tundra))
Caves and Subterranean		"cave" OR "caves" OR "subterranean"
Cryosphere		"cryosphere" OR "arctic" OR "antarctic" OR "polar" OR "subarctic" OR "subantarctic"
Desert and Xeric Shrublands		("desert" OR "deserts") OR (("xeric") AND ("habitat" OR "scrub*" OR "shrub*"))
Inland Waters	Bogs, Fens, Peatlands	"bog" OR "bogs" OR "fen" OR "fens" OR "mire" OR "peatland*" OR "pocosin*" OR "quagmire"
	Ephemeral Wetlands	"ephemeral wetland*" OR "ephemeral pool*" OR "temporary pond*" Or "vernal pool*" OR "vernal pond*"
	Estuaries & Deltas	"estuary" OR "estuaries" OR "inland delta*" OR "freshwater delta*" OR "river delta*" OR "tidal delta*"
	Floodplains	"flooded grassland*" OR "flooded savanna*" OR "floodplain*" OR "flood plain*"
	Lakes	"lake" OR "lakes"
	Marshes	"marsh" OR "marshes" OR "prairie pothole*"
	Rivers	"river" OR "rivers"
	Streams & Creeks	"stream" OR "streams" OR "creek" OR "creeks" OR "brook" OR "brooks"
	Swamps	"swamp" OR "swamps"
	Wetlands	wetland*
Mediterranean		("chaparral" OR "fynbo*" OR "kwongan*" OR "macchia" OR "maquis" OR "matorral") OR (("mediterranean") AND ("forest*" OR "woodland" OR "savanna*" OR "scrub*" OR "shrub*"))
Montane Grasslands & Shrublands		("*alpine" OR "montane") AND ("grassland*" OR "meadow*" OR "savanna*" OR "steppe* " OR "scrub*" OR "shrub*")
Shelf Ecosystems (Coastal Areas)	Beaches & Sand Dunes	"beach" OR "beaches" OR "sand bar*" OR "sand dune*" OR "sand* shore*"
	Coral Reefs	"coral reef*"
	Intertidal/Littoral Zone	"intertidal area*" OR "intertidal zone*" OR "littoral area*" OR "littoral zone*" OR "rock* reef*" OR "rock* shore*"
	Kelp Forests	kelp
	Lagoons & Saltmarshes	"lagoon*" OR "salt marsh*" OR "salt pond*" OR "tidal marsh*" OR "tidal pool*" OR "tidal pond*"
	Mangroves	mangrove*
	Seagrass	seagrass*
Surface Open Ocean & Deep Sea		"marine" OR "ocean" OR "oceans" OR "sea" OR "seas"
Temperate Grasslands		("pampa*" OR "prairie*") OR (("temperate") AND ("grassland*" OR "meadow*" OR "savanna*" OR "steppe*"))
Temperate/Boreal Forests/Woodlands		("boreal forest*" OR "deciduous forest*" OR "mixed forest*" OR "taiga") OR (("temperate") AND ("*forest*" OR "woodland*"))
Tropical/Subtropical Grasslands		("tropical" OR "subtropical") AND ("grassland*" OR "meadow*" OR "savanna*" OR "steppe*")
Tropical/Subtropical Forests/Woodlands		("tropical" OR "subtropical") AND ("*forest*" OR "woodland*")

**Anthromes**		
Aquaculture		"*culture pond*" OR "crab pond*" OR "fish pond*" OR "shrimp pond*"
Cultivated Areas		"agroecosystem*" OR "agro-ecosystem*" OR "agroforest" OR "agroforests" OR "agro-forest" OR "agro-forests" OR "cropland*" OR "orchard*" OR "paddy" OR "paddies" OR "pasture" OR "pastures" OR "pastureland*" OR "plantation*" OR "ranch" OR "ranches" OR "rangeland*"
Urban/Semi-Urban		"artificial lake" OR "artificial lakes" OR "artificial pond*" OR "artificial pond*" OR "artificial wetland*" OR "blue space*" OR "constructed wetland*" OR "detention pond*" OR "dike pond*" OR "drainage pond*" OR "green space*" OR "modified pond*" OR "rainwater*pond*" OR "reclamation pond*" OR "retention basin" OR "retention pond*" OR "sediment* basin*" OR "sediment* pond*" OR "settling basin" OR "settling pond*" OR "stormwater*pond*" OR "tailing pond*" OR "*urban area*" OR "*urban forest*" OR "urban pond*" OR "*urban wetland*" OR "wet pond*"
