# Supplementary material for: Research effort devoted to regulating and supporting ecosystem services by environmental scientists and economists
Source: PLoS One. 2021 May 28;16(5):e0252463. doi: 10.1371/journal.pone.0252463 (PMC8162671; doi:10.1371/journal.pone.0252463)
Supplement: S1 File — Description of how regulating and supporting ecosystem services as well as ecosystem types were selected. (PDF) [file pone.0252463.s003.pdf]

PLoS ONE

Electronic Supporting Information: S1 File

Title: **Research effort devoted to regulating and supporting ecosystem services by environmental scientists and economists**

Authors: Andrew N. Kadykalo, Lisa A. Kelly, Albana Berberi, Jessica L. Reid, C. Scott Findlay

S1 File. Selection of biophysical ecosystem services and ecosystem types. Description of how regulating and supporting ecosystem services as well as ecosystem types were selected.

Selection of Biophysical Ecosystem Services

We began with an initial set of 13 biophysical regulating and supporting ecosystem services based on the typology presented in the Value of Nature to Canadians Study Taskforce (2017), itself a revision of the typologies developed by De Groot et al. (2002), Millennium Ecosystem Assessment (2005), The Economics of Ecosystems and Biodiversity Project (2010), and Landsberg et al. (2013): regulating (air-quality regulation, climate regulation and carbon sequestration, water-flow regulation, erosion regulation, water purification and waste treatment, disease regulation, pest regulation, pollination, natural hazard regulation); supporting (soil formation, nutrient cycling, water cycling, habitat provision). From this list, we eliminated 'climate regulation' because the set of ecosystem functions which might affect the level of such service was deemed too large and poorly defined. As a substitute, we used the more specific service 'carbon sequestration'. As 'water cycling' was deemed not sufficiently distinct from 'water-flow regulation' the two were combined into one category ('water regulation'). 'Natural hazard regulation' was partitioned into three more specific processes and functions ('coastal and storm protection', 'drought mitigation', and 'flood regulation'). We also separated 'seed dispersal' from 'pollination', as the former function is solely concerned with the spreading or seeds and spores while the latter function is also concerned with fertilization (see CICES V5.1, Haines-Young and Potschin 2018). Several other potential biophysical ecosystem services were explored in preliminary searches ('avalanche control', 'carrion/scavenging services', 'fire prevention', 'landslide regulation', 'noise regulation', 'temperature regulation') but resulted in few hits (< 20 total). The fifteen biophysical ecosystem services considered in the analysis (S1 Table) represent those for which service delivery depends on a reasonably well-characterized set of ecological processes and functions with properties that can – at least in principle – be measured or estimated.

Selection of Ecosystem Types

We employed an ecosystem classification system similar to the 'Units of Analysis' classification system of the IPBES Global Assessment on Biodiversity and Ecosystem Services (IPBES 2019a; IPBES 2019b). This classification system includes 'Biomes' or 'Ecoregions' and 'Anthromes' (anthropogenic biomes, classes where ecosystem structure and function have been severely altered through human management). We combined 'Surface Open Ocean' and 'Deep Sea' as bibliometric literature searches are unlikely to distinguish explicitly between the services provided by these two ecosystems. Moreover, we added two more classes identified in other classifications but missing from the IPBES classification system: 'Montane Grasslands and Shrublands' from the World Wildlife Fund classification scheme (Olson et al. 2001; WWF 2020), and 'Caves and Subterranean' from the IUCN's Habitats Classification Scheme (IUCN 2020). Finally, some comparatively broad ecosystem classes (e.g. arctic and mountain tundra, inland waters, and shelf ecosystems) were further partitioned into more highly resolved subclasses. In total, we defined 32 ecosystem classes (whether subsystems, biome/ecoregion, or anthromes) here referred to as 'ecosystem type' (S2 Table).

References

De Groot R, Wilson MA, Boumans RMJ. A typology for the classification, description and valuation of ecosystem functions, goods and services. Ecol Econ. 2002;41(3): 393-408. doi: 10.1016/s0921-8009(02)00089-7.

Díaz S, Pascual U, Stenseke M, Martín-López B, Watson RT, Molnár Z, et al. Assessing nature's contributions to people. Science. 2018;359(6373): 270-272. doi: 10.1126/science.aap8826.

Haines-Young R, Potschin MB. Common international classification of ecosystem services (CICES) V5.1 and guidance on the application of the revised structure; 2018. Available from: https://cices.eu/content/uploads/sites/8/2018/01/Guidance-V51-01012018.pdf.

IPBES. Chapter 1. Assessing a planet in transformation: Rationale and approach of the IPBES Global Assessment on Biodiversity and Ecosystem Services. Secretariat of the Intergovernmental Science-Policy Platform on Biodiversity and Ecosystem Services, Bonn, Germany; 2019a.

IPBES. Chapter 2. Status and trends - Nature. Secretariat of the Intergovernmental Science-Policy Platform on Biodiversity and Ecosystem Services, Bonn, Germany; 2019b.

IUCN. Habitats Classification Scheme (Version 3.1); 2020. Available from: https://www.iucnredlist.org/resources/habitat-classification-scheme

Landsberg F, Treweek J, Stickler MM, Henninger N, Venn O. Weaving Ecosystem Services into Impact Assessment: A Step-By-Step Method. Washington DC: World Resources Institute; 2013.

Millennium Ecosystem Assessment.Ecosystems and Human Well-Being: Synthesis. Washington, DC; 2005.

Olson DM, Dinerstein E, Wikramanayake ED, Burgess ND, Powell GVN, Underwood EC, et al. Terrestrial Ecoregions of the World: A New Map of Life on Earth. BioScience. 2001;51(11): 933-938. doi: 10.1641/0006-3568(2001)051[0933:TEOTWA]2.0.CO;2.

The Economics of Ecosystems and Biodiversity Project. Mainstreaming the Economics of Nature: A Synthesis of the Approach, Conclusions and Recommendations of TEEB. London and Washington; 2010.

Value of Nature to Canadians Study Taskforce. Completing and Using Ecosystem Service Assessment for Decision-Making: An Interdisciplinary Toolkit for Managers and Analysts. Provincial, Federal, and Territorial Governments of Canada, Ottawa, ON; 2017. Avialable from: http://biodivcanada.ca/default.asp?lang=En&n=B443A05E-1

WWF. Ecoregions; 2020. Avaialble from: https://www.worldwildlife.org/biomes
