# Supplementary material for: Research effort devoted to regulating and supporting ecosystem services by environmental scientists and economists
Source: PLoS One. 2021 May 28;16(5):e0252463. doi: 10.1371/journal.pone.0252463 (PMC8162671; doi:10.1371/journal.pone.0252463)
Supplement: S2 File — The Number of Article Hits–Scopus; S2.1 Fig in S2 File. Variation among ecosystem types and ecosystem services (Scopus); Correlational Analysis–Scopus (S2.2 Fig in S2 File. Correlation between environmental science and economic valuation article hits); S2.3 Fig in S2 File. Research effort in environmental science and economic valuation (Scopus); S2.4 Fig in S2 File. Research effort differential between environmental science and economic valuation (Scopus). (PDF) [file pone.0252463.s004.pdf]

PLoS ONE

Electronic Supporting Information: S2 File

Title: **Research effort devoted to regulating and supporting ecosystem services by environmental scientists and economists**

Authors: Andrew N. Kadykalo, Lisa A. Kelly, Albana Berberi, Jessica L. Reid, C. Scott Findlay

S2 File. Scopus-specific results.

The Number of Article Hits – Scopus

Averaged over the $N = 480$ ecosystem type × ecosystem service combinations, the number of article hits was far greater in environmental sciences (14.6 ± 35.9 (1 SD) versus 1.9 ± 4.6 (1 SD) for economic valuation).

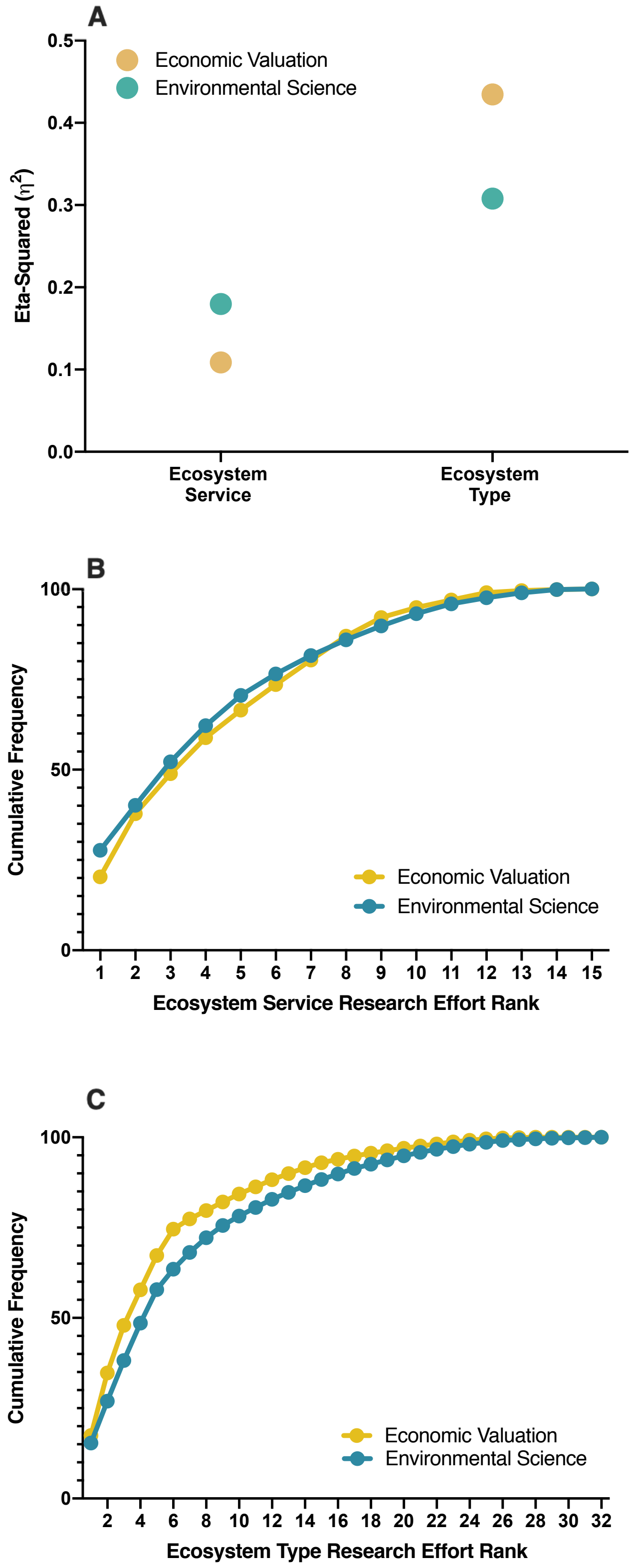

Fig S2.1. Variation among ecosystem types and ecosystem services (Scopus). (A) Eta-squared (η^2) for a two-factor (ecosystem service, ecosystem type) ANOVA of the number of article hits in the two research domains. Also shown are cumulative frequency plots for $N = 15$ ecosystem services (B) and $N = 32$ ecosystem types (C) in Scopus. See Kadykalo_etal_ESRE_data_6.csv for cumulative frequency data.

Correlational Analysis – Scopus

There was a moderately strong ($r = 0.66$, Fig S2.2A) correlation between research effort in the two domains based on the number of article hits for each of the ecosystem type × ecosystem service combinations. Focusing solely on ecosystem services (i.e. correlation based on N = 15 ecosystem services) or ecosystem types (i.e. correlation based on N = 32 ecosystem types) improved the correlation ($r = 0.68$ (Fig S2.2B) and 0.90 (Fig S2.2C), respectively) between research efforts in the two domains.

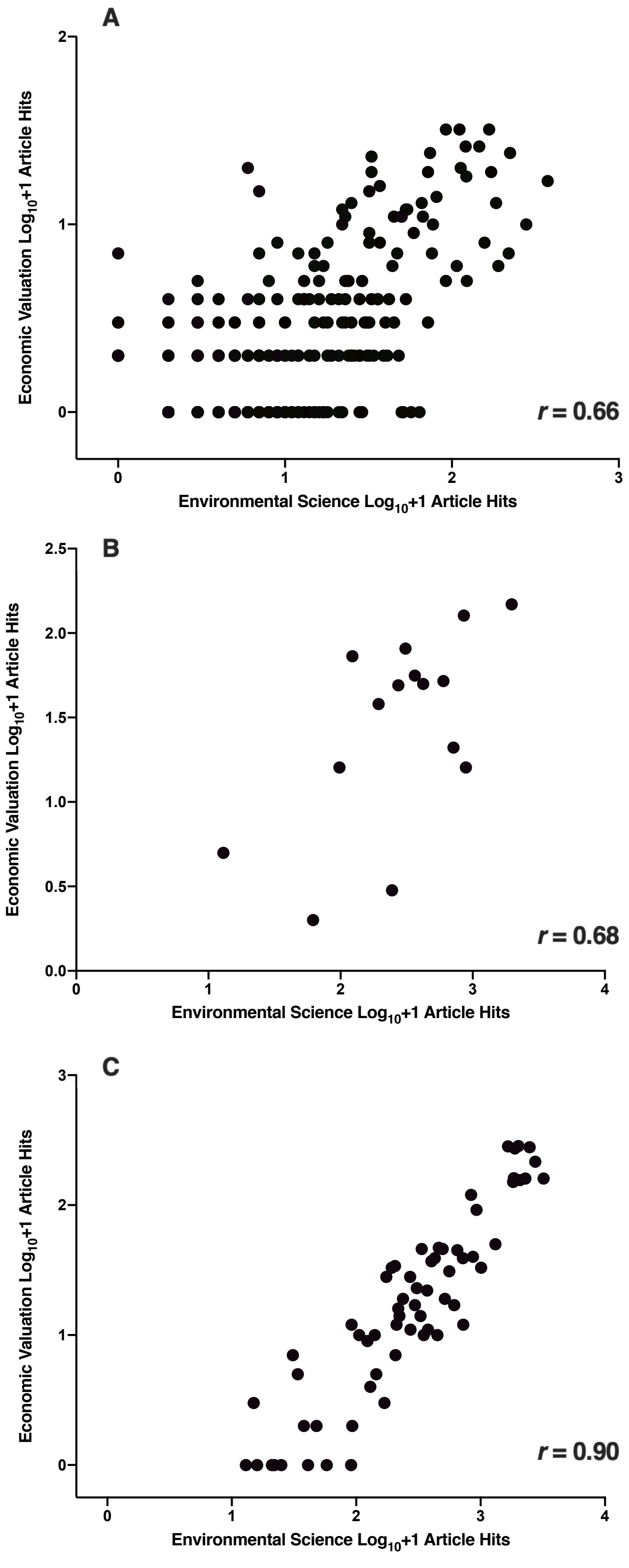

Fig S2.2. Correlation between environmental science and economic valuation article hits (Scopus). Scatterplot between environmental science and economic valuation research effort as estimated by the $\log_{10}+1$ number retrieved article hits for $N = 15$ biophysical ecosystem services × $N = 32$ ecosystem types (A), $N = 15$ individual biophysical ecosystem services (B), and $N = 32$ individual ecosystem types (C) in Scopus.

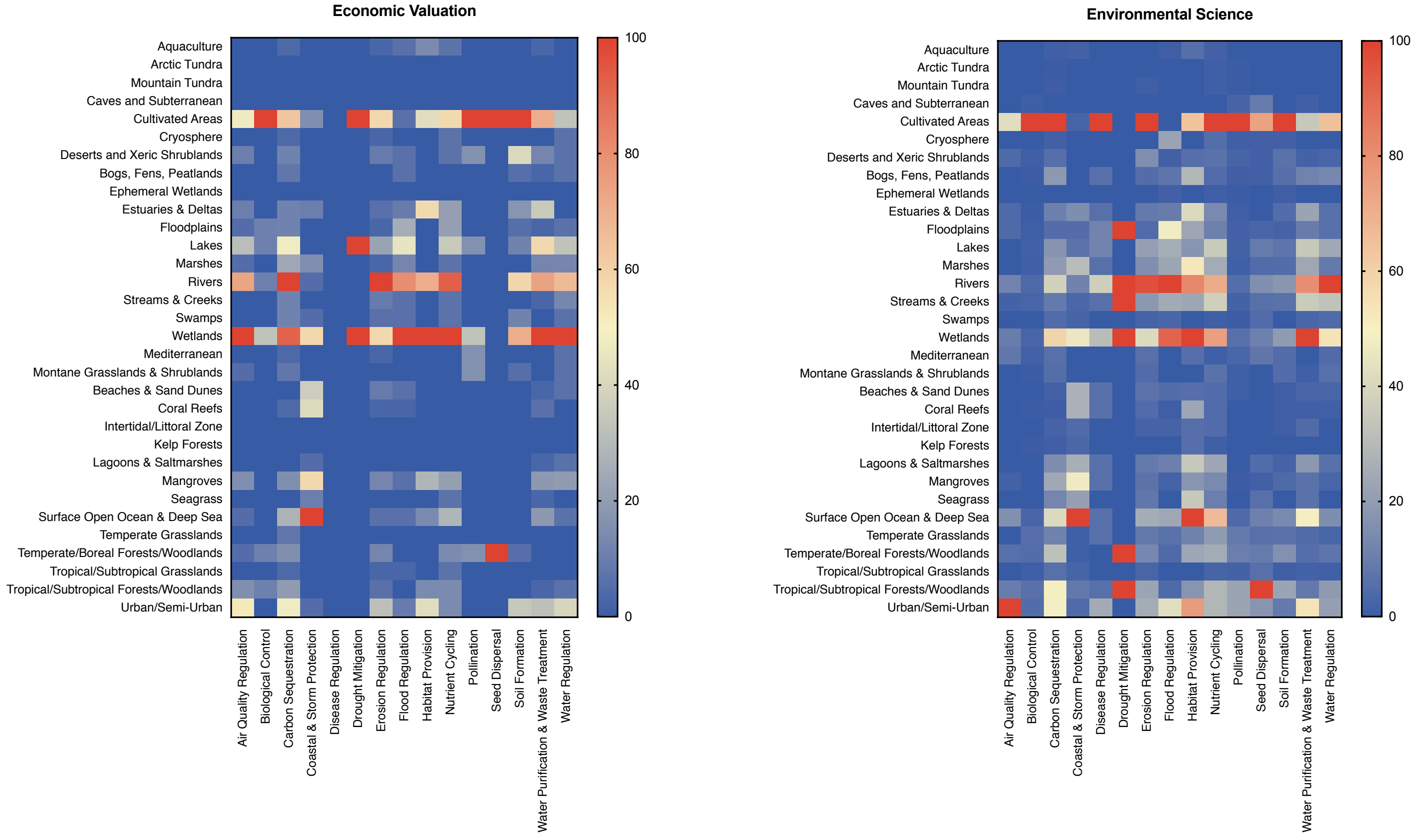

Fig S2.3. Research effort in environmental science and economic valuation (Scopus). Heat map of economic valuation and environmental science research effort on each of $N = 15$ biophysical ecosystem service and $N = 32$ ecosystem type combinations as estimated by retrieved article hits in Scopus. Raw article hits were normalized based on the smallest (0%) and largest (100%) values in each data set to allow for direct and relative comparison between research domains with respect to relative article hits. Red cells indicate higher, blue cells indicate lower research effort for that combination of ecosystem service and ecosystem type relative to the average effort.

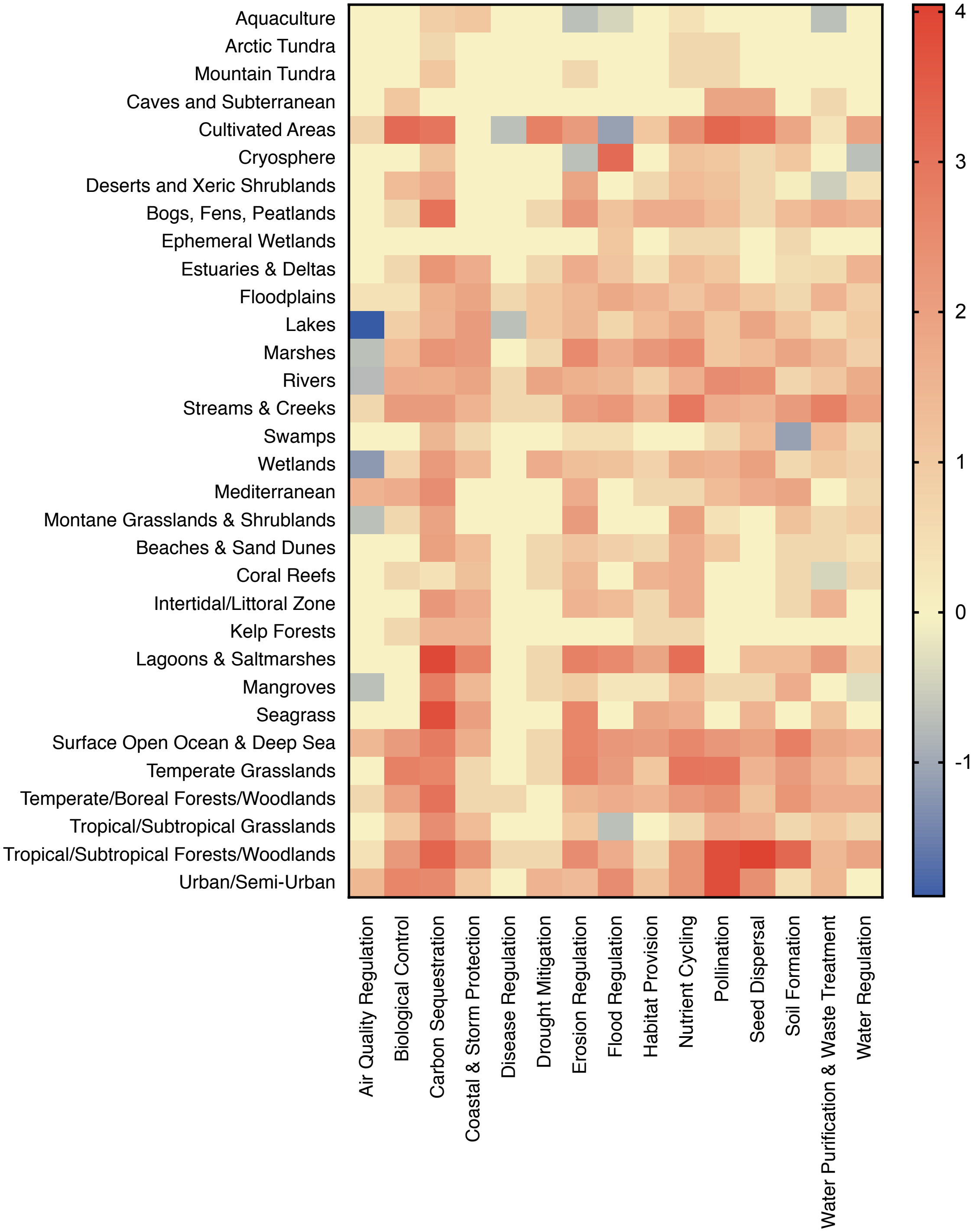

Fig S2.4. Research effort differential between environmental science and economic valuation (Scopus). Heat map of the z-transformed $Log_{10}+1$ research effort differential between environmental science and economic valuation research effort on each of $N = 15$ biophysical ecosystem service and $N = 32$ ecosystem type combinations as estimated by retrieved article hits in Scopus. Z-transformed differentials were normalized using the z-transformed value of the raw differential of zero (-0.97) to allow for direct comparison between research domains with respect to relative article hits. Red cells indicate higher environmental sciences, blue cells indicate higher economic valuation research effort for that combination of ecosystem service relative to the average effort. See Kadykalo_etal_ESRE_data_7.tab for research effort differential data.
