# Supplementary material for: Research effort devoted to regulating and supporting ecosystem services by environmental scientists and economists
Source: PLoS One. 2021 May 28;16(5):e0252463. doi: 10.1371/journal.pone.0252463 (PMC8162671; doi:10.1371/journal.pone.0252463)
Supplement: S3 File — Correlational Analysis–Web of Science vs. Scopus; Fig 3.1 in S3 File. Correlation between environmental science and economic valuation article hits for each of the 15 biophysical ecosystem services; S3.1 Table in S3 File. Pearson correlation coefficients between economic valuation and environmental science research effort on 15 selected biophysical ecosystem services; S3.2 Table in S3 File. Pearson correlation coefficients between economic valuation and environmental science research effort on 32 selected ecosystem types; S3.3 Table in S3 File. The total and mean number of retrieved article hits in Web of Science and Scopus for environmental science and economic valuation research effort on 15 biophysical ecosystem services; S3.2 Fig in S3 File. Bar graph of economic valuation and environmental science research effort on N = 15 selected biophysical ecosystem services, pooling over all potential ecosystem types; S3.4 Table in S3 File. The total and mean number of retrieved article hits in Web of Science and Scopus for environmental science and economic valuation research effort on 32 ecosystem types; S3.3 Fig in S3 File. Bar graph of economic valuation and environmental science research effort on each of N = 32 ecosystem types, pooling over all potential ecosystem services). (PDF) [file pone.0252463.s005.pdf]

PLoS ONE

Electronic Supporting Information: S3 File

Title: **Research effort devoted to regulating and supporting ecosystem services by environmental scientists and economists**

Authors: Andrew N. Kadykalo, Lisa A. Kelly, Albana Berberi, Jessica L. Reid, C. Scott Findlay

S3 File. Additional results.

Correlational Analysis – Web of Science vs. Scopus

The number of environmental science hits was highly correlated between Web of Science and Scopus databases ($r = 0.98$, pooled over services); the correlation was somewhat smaller ($r = 0.89$) for economic valuation hits.

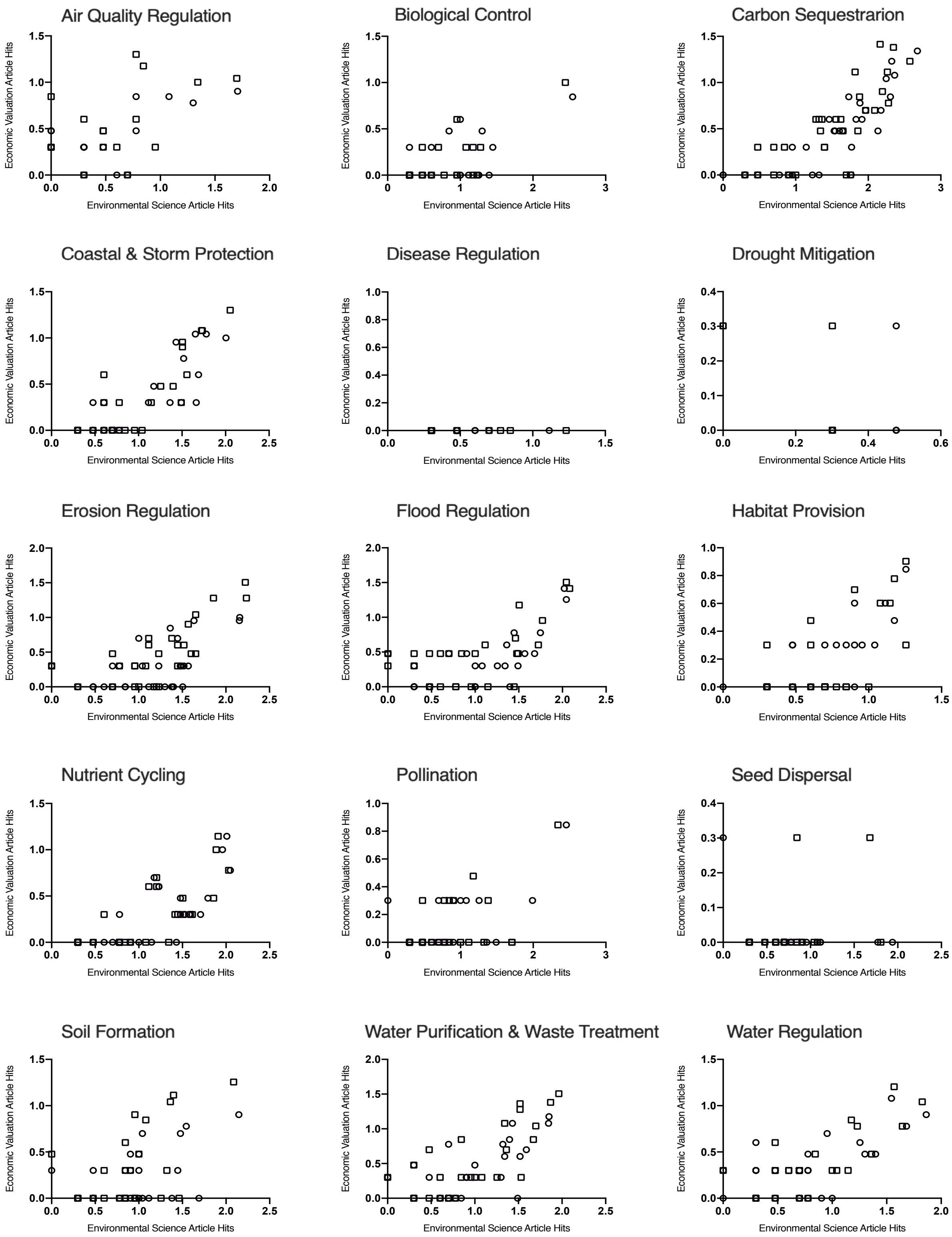

Fig S3.1. Correlation between environmental science and economic valuation article hits for each of the 15 biophysical ecosystem services. The correlation between environmental science and economic valuation research effort on 15 biophysical ecosystem services as estimated by retrieved article hits ($\log_{10}$+1) in Scopus (hollow squares) and Web of Science (hollow circles). Each datum in the figure gives the number of retrieved hits for each of $N = 32$ possible ecosystem types.

Table S3.1. Pearson correlation coefficients between economic valuation and environmental science research effort on 15 selected biophysical ecosystem services. Based on retrieved article hits in Web of Science and Scopus. Correlation based on *N* = 32 ecosystem types.

**Ecosystem Service**	**Pearson Correlation**	
	**Web of Science**	**Scopus**
Air Quality Regulation	0.75	0.50
Biological Control	0.82	0.94
Carbon Sequestration	0.92	0.75
Coastal & Storm Protection	0.81	0.95
Disease Regulation	N/C	N/C
Drought Mitigation	N/C	N/C
Erosion Regulation	0.78	0.91
Flood Regulation	0.89	0.90
Habitat Provision	0.84	0.74
Nutrient Cycling	0.80	0.83
Pollination	0.92	0.87
Seed Dispersal	N/C	N/C
Soil Formation	0.72	0.78
Water Purification & Waste Treatment	0.83	0.87
Water Regulation	0.77	0.81

'N/C' = Not calculable due to low sample sizes

Table S3.2. Pearson correlation coefficients between economic valuation and environmental science research effort on 32 selected ecosystem types. Based on retrieved article hits in Web of Science and Scopus. Correlation based on $N = 15$ ecosystem services.

Ecosystem Type	Pearson Correlation	
	Web of Science	Scopus
Aquaculture	N/C	0.41
Arctic Tundra	N/C	N/C
Mountain Tundra	N/C	N/C
Caves and Subterranean	N/C	N/C
Cultivated Areas	0.73	0.47
Cryosphere	N/C	N/C
Deserts and Xeric Shrublands	0.38	0.45
Bogs, Fens, Peatlands	0.92	0.68
Ephemeral Wetlands	N/C	N/C
Estuaries & Deltas	0.73	0.52
Floodplains	0.86	0.96
Lakes	0.44	0.73
Marshes	0.79	0.85
Rivers	0.79	0.91
Streams & Creeks	0.68	0.82
Swamps	0.58	0.70
Wetlands	0.77	0.70
Mediterranean	0.75	0.67
Montane Grasslands & Shrublands	N/C	0.68
Beaches & Sand Dunes	0.74	0.95
Coral Reefs	0.90	0.93
Intertidal/Littoral Zone	N/C	N/C
Kelp Forests	N/C	N/C
Lagoons & Saltmarshes	0.93	N/C
Mangroves	0.73	0.56
Seagrass	N/C	N/C
Surface Open Ocean & Deep Sea	0.76	0.77
Temperate Grasslands	N/C	N/C
Temperate/Boreal Forests/Woodlands	0.82	0.77
Tropical/Subtropical Grasslands	0.47	0.49
Tropical/Subtropical Forests/Woodlands	0.79	0.67
Urban/Semi-Urban	0.70	0.64

'N/C' = Not calculable due to low sample sizes

Table S3.3. The total and mean number of retrieved article hits in Web of Science and Scopus for environmental science and economic valuation research effort on 15 biophysical ecosystem services. The total of reported hits is pooled over all potential ecosystem types (i.e. each hit is unique) while the mean of reported hits is averaged over $N = 32$ possible ecosystem types.

Ecosystem Service	Environmental Science				Economic Valuation			
	Web of Science		Scopus		Web of Science		Scopus	
	Total	Mean	Total	Mean	Total	Mean	Total	Mean
Air Quality Regulation	105	3.25	122	3.41	33	1.19	72	2.28
Biological Control	879	16.25	714	12.53	20	0.50	20	0.53
Carbon Sequestration	2059	73.78	1973	65.06	131	3.34	147	4.38
Coastal & Storm Protection	185	14.56	193	14.16	26	1.66	37	2.19
Disease Regulation	44	1.06	61	1.44	1	0	1	0
Drought Mitigation	15	0.38	12	0.19	2	0.03	4	0.09
Erosion Regulation	621	21.25	857	25.72	46	1.50	126	3.63
Flood Regulation	330	17.31	364	17.47	46	2.22	55	3.38
Habitat Provision	90	3.78	97	4.31	13	0.69	15	0.84
Nutrient Cycling	573	23.44	599	21.16	28	1.50	51	2.09
Pollination	1036	16.66	887	14.13	20	0.41	15	0.41
Seed Dispersal	271	7.91	244	6.22	2	0.03	2	0.06
Soil Formation	467	13.28	421	10.16	29	0.94	49	1.97
Water Purification & Waste Treatment	241	12.53	309	15.06	35	2.25	80	4.53
Water Regulation	257	8.75	273	8.50	14	1.47	48	1.84

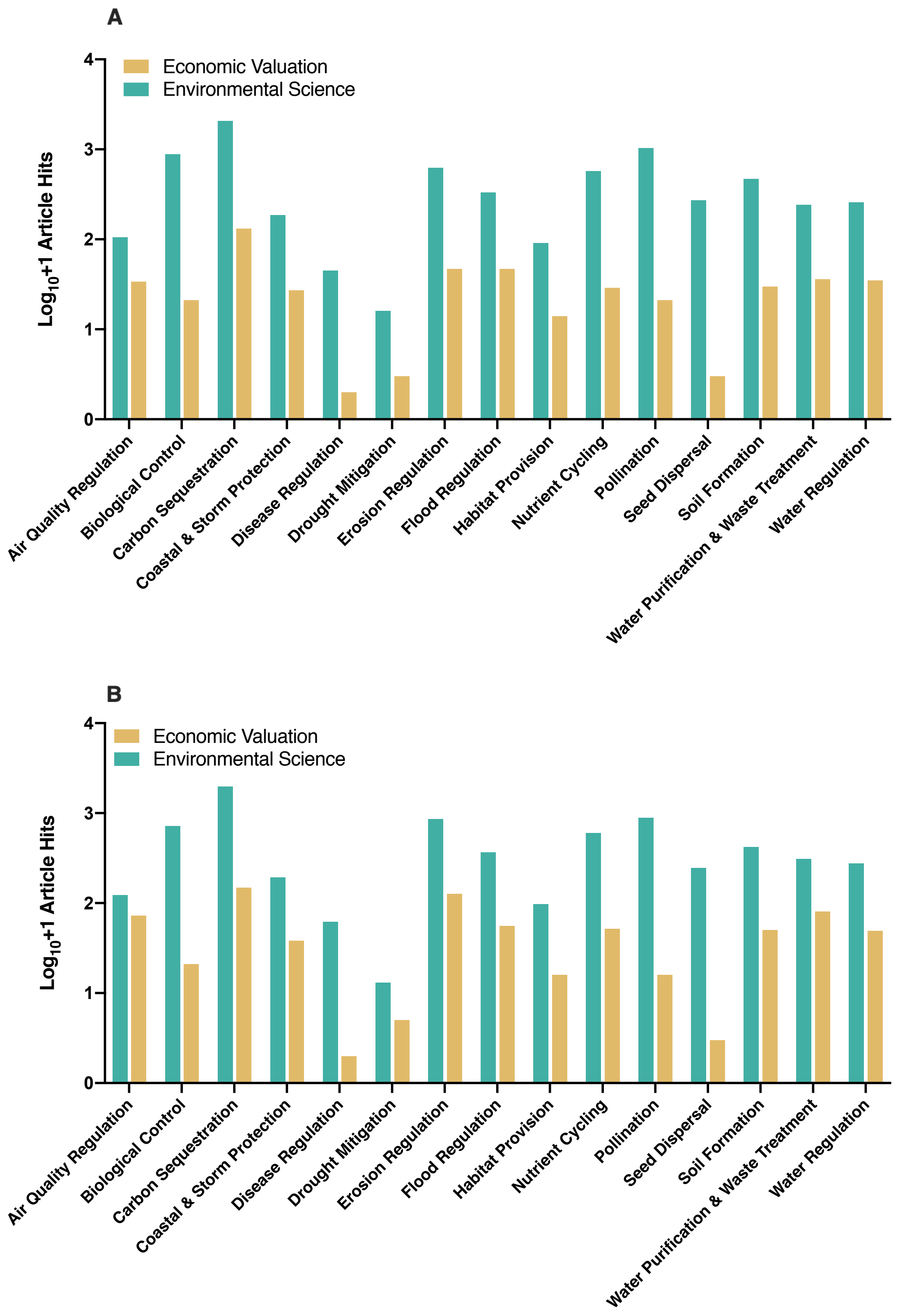

Fig S3.2. Bar graph of economic valuation and environmental science research effort on $N = 15$ selected biophysical ecosystem services, pooling over all potential ecosystem types. As estimated by retrieved absolute $\log_{10}+1$ article hits in Web of Science (A) and Scopus (B).

Table S3.4. The total and mean number of retrieved article hits in Web of Science and Scopus for environmental science and economic valuation research effort on 32 ecosystem types. The total of reported hits is pooled over all potential ecosystem services (i.e. each hit is unique) while the mean of reported hits is averaged over $N = 15$ possible ecosystem services.

**Ecosystem Type**		**Environmental Science**				**Economic Valuation**			
**Biomes/Ecoregions**	**Subsystem**	**Web of Science**		**Scopus**		**Web of Science**		**Scopus**	
		**Total**	**Mean**	**Total**	**Mean**	**Total**	**Mean**	**Total**	**Mean**
Arctic and Mountain Tundra	Arctic Tundra	21	0.27	12	0.20	0	0.00	0	0.00
	Mountain Tundra	15	0.20	14	0.33	0	0.00	2	0.00
Caves and Subterranean		47	0.73	40	1.00	1	0.00	0	0.00
Cryosphere		129	1.27	122	2.93	3	0.07	8	0.27
Desert and Xeric Shrublands		237	6.20	204	5.53	18	0.73	33	1.60
Inland Waters	Bogs, Fens, Peatlands	222	7.67	210	8.80	13	0.40	11	0.47
	Ephemeral Wetlands	24	0.33	20	0.33	0	0.00	0	0.00
	Estuaries & Deltas	650	13.07	460	9.67	44	1.53	46	2.20
	Floodplains	371	10.00	306	9.13	21	1.20	22	1.33
	Lakes	928	12.47	838	17.00	91	2.00	119	4.80
	Marshes	560	19.20	402	16.27	30	1.00	36	1.47
	Rivers	2480	52.27	2012	49.40	278	5.47	284	10.53
	Streams & Creeks	867	16.40	720	15.20	39	0.53	38	0.80
	Swamps	105	2.27	91	2.67	9	0.33	11	0.80
	Wetlands	1883	45.60	1659	49.53	272	7.80	282	12.47
Mediterranean		296	6.80	217	4.47	16	0.40	15	0.27
Montane Grasslands & Shrublands		168	3.40	140	3.33	2	0.00	9	0.40
Shelf Ecosystems (Coastal Areas)	Beaches & Sand Dunes	192	5.00	174	4.73	32	0.80	27	0.93
	Coral Reefs	336	3.47	271	3.73	45	0.93	27	0.87
	Intertidal/Littoral Zone	92	1.60	90	2.13	1	0.00	0	0.00
	Kelp Forests	57	0.80	37	0.73	0	0.00	1	0.00
	Lagoons & Saltmarshes	515	16.60	328	11.60	18	0.60	13	0.20
	Mangroves	495	12.47	428	12.93	45	2.20	38	2.53
	Seagrass	377	9.60	272	7.60	10	0.07	10	0.27
Surface Open Ocean & Deep Sea		2292	31.93	2072	35.67	159	1.53	155	2.87
Temperate Grasslands		449	12.07	349	9.73	9	0.07	9	0.13
Temperate/Boreal Forests/Woodlands		726	17.80	614	17.27	11	0.27	16	1.00
Tropical/Subtropical Grasslands		206	5.07	144	3.33	6	0.67	4	0.27
Tropical/Subtropical Forests/Woodlands		1317	40.87	1009	30.93	49	1.67	32	1.07
**Anthromes**									
Aquaculture		33	0.40	30	0.67	4	0.20	6	0.47
Cultivated Areas		3214	113.67	2749	96.07	159	5.40	215	8.00
Urban/Semi-Urban		1824	30.13	1849	35.33	150	1.93	160	4.20

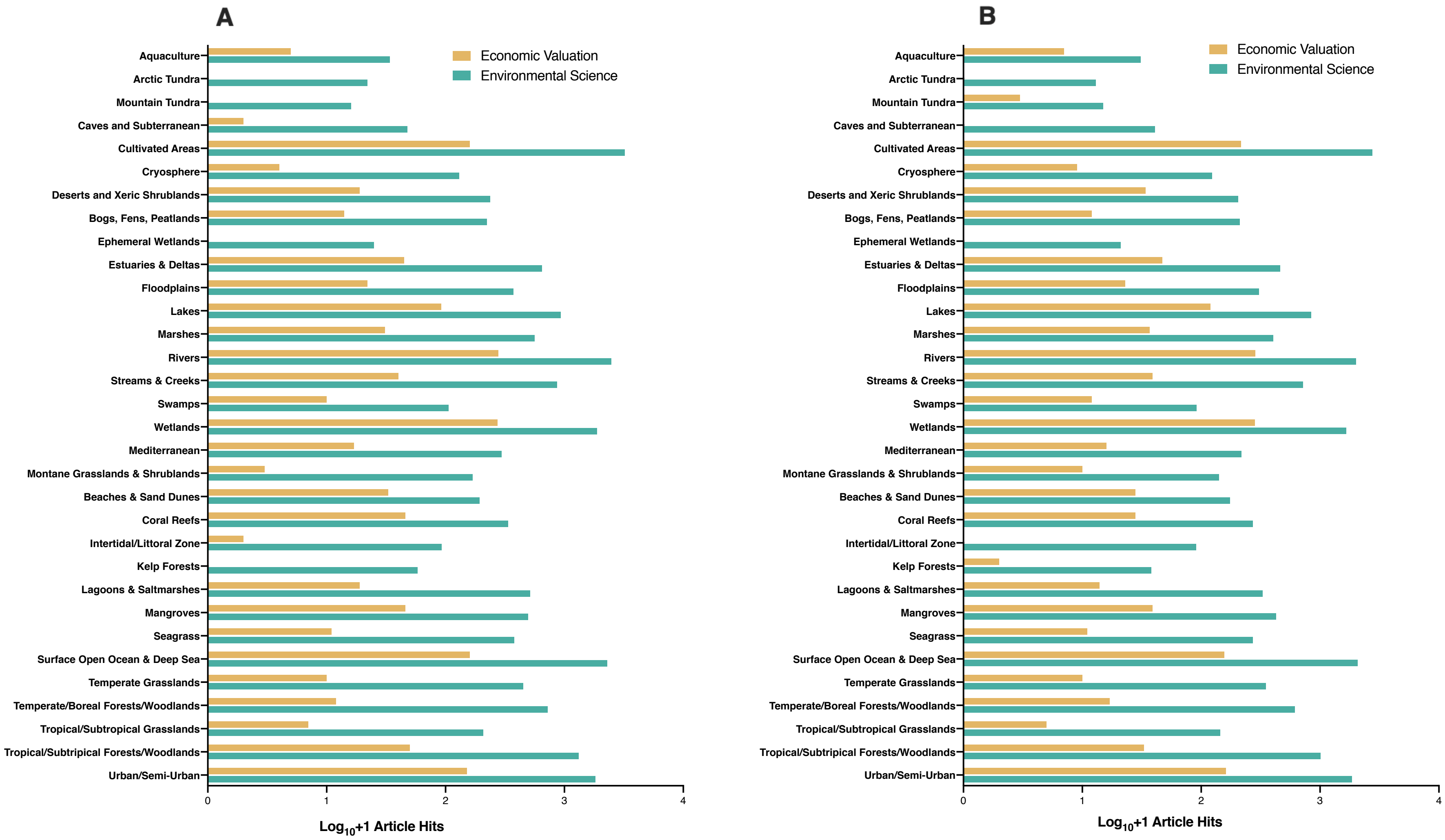

Fig S3.3. Bar graph of economic valuation and environmental science research effort on each of $N = 32$ ecosystem types, pooling over all potential ecosystem services. As estimated by retrieved absolute $\log_{10}$+1 article hits in Web of Science (A) and Scopus (B).
