# Supplementary material for: Research effort devoted to regulating and supporting ecosystem services by environmental scientists and economists
Source: PLoS One. 2021 May 28;16(5):e0252463. doi: 10.1371/journal.pone.0252463 (PMC8162671; doi:10.1371/journal.pone.0252463)
Supplement: Attachment — Submitted filename: Review_PONE-D-21-00613.pdf [file pone.0252463.s006.pdf]

Review report on manuscript PONE D-21-00613

“Research effort devoted to regulating and supporting ecosystem services by environmental scientists and economists”

The paper presents a bibliographical analysis of research done in the domain of ecosystem services, focusing on environmental science aspects on the one hand, and on economic valuation, on the other. For the main part, the paper contrasts the frequencies at which different types of ecosystems and different types of ecosystem services have been studied in one or the other field. As the literature is expanding, an attempt to quantitatively analyze and structure this literature is in principle welcome. Yet, the analysis in the present paper remains largely descriptive, and provides little analytical insight.

In the following I list my specific questions, comments, and concerns.

1. I do not agree that valuation always requires to quantify ecosystem services in physical units. For the so-called ‘cultural ecosystem services’ this is quite obvious. For example, the value of recreational benefit can be measured in in monetary units, whereas I have no idea how environmental scientists could physically quantify recreational services without somehow resorting to values. As another example, tn the list of NCPs there is the ‘maintenance of options’. This is also one example where valuation is possible, but physical quantification hard to imagine. Against this background, a solid argument is needed why physical quantification is a prerequisite for valuation when it comes to regulating and supporting ecosystem services. As it stands, this is merely an unsupported claim that is repeatedly made in the paper.
2. That said, it is required to know how ecosystems are functioning if the aim is to make a valuation study useful for decision-making. Otherwise it would not be possible to assess how management changes would change the value of ecosystem services. So my previous remark does not invalidate the claim that an integrated research agenda is desirable.

3. The measure of research effort devoted to certain types of ecosystem services or types of ecosystems should somehow be normalized. One of the reasons why cultivated areas are more researched than kelp forest ecosystems probably is the much larger area covered by the former.
4. For this particular paper it might be useful to mention the disciplinary background of the authors: How many of them are economists, how many are environmental scientists?
5. I do not generally object the search strings applied. However, more needs to be said on the method how they were generated. As such, the choice of the search string merely seems to appeal to plausibility.
6. Figure 2. Why is the data not presented on a linear scale?
7. Figures 3/4. I do not easily get the analytical message that these heat maps are supposed to convey. The seem to be purely descriptive to me.
8. Is there any evidence that supports the hypotheses why economists and environmental scientists choose their respective focus?
9. The implications for ecosystem management and decision-making seem not supported by the analysis. In particular the fact that "there exists a corpus of relevant economic valuation work" was known before.
10. I agree that the "results provide little insight into the extent to which environmental scientists and economists are engaged in joint research enterprises". Thus, also no conclusions should be drawn in this regard.
11. The correlation of research in environmental science and valuation may be driven by societal demand, which should be welcome from the decision-maker's point of view.
